# TAp73 promotes cell survival upon genotoxic stress by inhibiting p53 activity

**DOI:** 10.18632/oncotarget.2440

**Published:** 2014-09-22

**Authors:** Dongshi Chen, Lihua Ming, Fangdong Zou, Ye Peng, Bennett Van Houten, Jian Yu, Lin Zhang

**Affiliations:** ^1^ University of Pittsburgh Cancer Institute, Pittsburgh, PA, 15213, USA; ^2^ Department of Pharmacology and Chemical Biology, University of Pittsburgh School of Medicine, Pittsburgh, PA, 15213, USA; ^3^ Department of Pathology, University of Pittsburgh School of Medicine, Pittsburgh, PA, 15213, USA; ^4^ College of Life Sciences, Sichuan University, Chengdu, Sichuan, 610064, P.R. China

## Abstract

p53 plays a key role in regulating DNA damage response by suppressing cell cycle progression or inducing apoptosis depending on extent of DNA damage. However, it is not clear why mild genotoxic stress favors growth arrest, whereas excessive lesions signal cells to die. Here we showed that TAp73, a p53 homologue thought to have a similar function as p53, restrains the transcriptional activity of p53 and prevents excessive activation of its downstream targets upon low levels of DNA damage, which results in cell cycle arrest. Extensive DNA damage triggers TAp73 depletion through ubiquitin/proteasome-mediated degradation of E2F1, leading to enhanced transcriptional activation by p53 and subsequent induction of apoptosis. These findings provide novel insights into the regulation of p53 function and suggest that TAp73 keeps p53 activity in check in regulating cell fate decisions upon genotoxic stress.

## INTRODUCTION

Mammalian cells have developed an intricate molecular network to deal with DNA damage inflicted by frequent environmental or endogenous insults [1]. Depending on various factors, DNA damage can trigger DNA repair, cell cycle arrest, or apoptosis [2]. The central regulator of DNA damage response is the tumor suppressor p53, which either inhibits cell growth by activating p21, 14-3-3σ, and other cell cycle regulators, or induces apoptosis through proapoptotic targets such as PUMA, Noxa and Bax [3]. DNA damage response is essential for maintenance of genomic integrity and functions as a guardian against oncogenic transformation [4]. Tumor cells are almost invariably defective in DNA damage response due to defects in the p53 and other DNA repair pathways [4]. Furthermore, ionizing radiation and chemotherapeutic drugs used in anticancer therapies often kill tumor cells by inducing toxic levels of DNA damage [5].

In addition to p53, several other p53 family members, such as p73 and p63, also play a significant role in DNA damage response [6]. p73 is expressed in two major isoform classes, including TAp73 and ΔNp73, which have distinct functions [7]. Similar to p53, TAp73 isoforms contain highly conserved DNA binding, transactivation, and oligomerization domains, whereas ΔNp73 lacks the transactivation domain but contains DNA-binding and oligomerization domains [7]. Following DNA damage, TAp73 can bind to the same set of p53-responsive elements and activate p53 target genes to arrest cell cycle or induce apoptosis [8]. Although TAp73 was shown to be a tumor suppressor [9, 10], it is rarely mutated in human tumors [11], and p73-deficient mice do not resemble p53-null mice in tumor phenotypes [9, 12]. Unlike p53, which is consistently proapoptotic, TAp73 can be proapoptotic or antiapoptotic [13, 14]. TAp73 expression can be either upregulated or downregulated in response to different DNA damaging agents [15]. These observations suggest that the function of p73 does not overlap with that of p53 in DNA damage response.

A fundamental and unresolved issue is how cells respond to different levels of stress. It is unclear why transient or low levels of DNA damage suppress cell growth, but extensive and persistent lesions often lead to apoptosis. Recent studies indicate that specific events can be triggered by excessive DNA damage to alert neighboring cells, or to eliminate the damaged cells by apoptosis [16]. However, little is known about how p53 activity is adjusted in response to different stress levels. In this study, we uncovered a function of TAp73 in restraining p53 activity in response to low levels of DNA damage. In the context of extensive DNA damage, depletion of TAp73 leads to enhanced proapoptotic activities of p53. Our results provide insight into cell fate determination through the interplay of p53 family members.

## RESULTS

### Downregulation of TAp73 following extensive DNA damage

There are at least 30 *p73* transcript isoforms generated by two different promoters (TA and ΔN) and extensive alternative slicing. TAp73α is the most prominent and transcriptionally competent p73 isoform that resembles p53 [7]. To distinguish TAp73 from ΔNp73, a triple-Flag tag (3×Flag) was knocked into the N-terminus of TAp73 in *p53*-wildtype (WT) HCT116 colon cancer cells (Fig. S1, A and B). TAp73α (TAp73 hereafter) in the Flag-knock-in (Flag-KI) cells could be specifically detected by Flag western blotting (Fig. S1C). No other TAp73 isoforms, such as TAp73β, could be detected in HCT116 cells (Fig. S1, D and E). Knock-in of the Flag tag does not affect the sensitivity of HCT116 cells to DNA damage-induced apoptosis (Fig. S1F).

**Figure 1:**
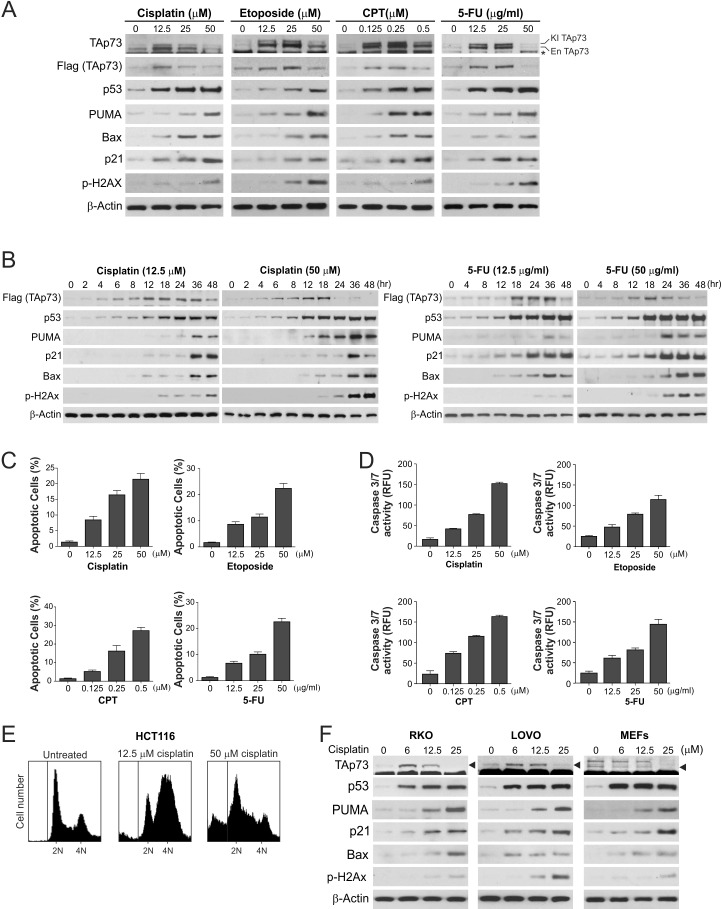
Downregulation of TAp73 in response to extensive DNA damage correlates with induction of apoptosis **(A)** HCT116 human colon cancer cells with knock-in (KI) of Flag-tagged TAp73 were treated with cisplatin, etoposide, camptothecin (CPT), or 5-fluorouracil (5-FU) at indicated concentrations for 24 hr. Indicated proteins were analyzed by western blotting. KI TAp73 and En TAp73 indicate knock-in and endogenous forms of TAp73α, respectively. * indicates a non-specific band detected by the p73 antibody. **(B)** Time course of expression of indicated proteins in HCT116 cells treated with cisplatin (12.5 or 50 μM) or 5-FU (12.5 or 50 μg/ml) was analyzed by western blotting. **(C)** Following treatment of cells as in (A) for 24 hr, apoptosis was analyzed by counting cells with condensed and fragmented nuclei following staining with Hoechst 33258. **(D)** Caspase activity in HCT116 cells treated as in (A) for 24 hr was measured using the caspase-3/7 assay kit, as described in the Materials and Methods. Results were expressed as means ± s.d. of three independent experiments. **(E)** HCT116 cells were treated with cisplatin as indicated for 24 hr. Cell cycle was analyzed by propidium iodide (PI) staining followed by flow cytometry. **(F)** RKO, LOVO and MEF cells were treated with cisplatin at indicated concentrations for 24 hr. Indicated proteins were analyzed by western blotting. Arrowheads indicate TAp73 bands.

To investigate the role of TAp73 in DNA damage response, Flag-KI cells were treated with several DNA damaging agents at various concentrations. Both knock-in and endogenous forms of TAp73 were induced by a 24-hr treatment with either 12.5 μM cisplatin, 12.5 μM etoposide, 0.125 μM camptothecin (CPT), or 12.5 μg/ml 5-Fluorouracil (5-FU) (Fig. 1A, S1D and S1E), and TAp73 accumulation was detected through 36 hr after treatment with 12.5 μM cisplatin (Fig. 1B). These conditions induced relatively low levels of DNA damage indicated by histone 2Ax phosphorylation (γH2Ax), modest and delayed (after 24 hr) substantial induction of p53 target genes (Fig. 1A and 1B), and predominantly cell cycle arrest, but little apoptosis or caspase activation (Fig. 1, C-E). Treating cells with DNA damaging agents at escalated doses, including 50 μM cisplatin, 50 μM etoposide, 0.5 μM CPT, and 50 μg/ml 5-FU, led to higher levels of DNA damage, p53, and its target genes (Fig. 1, A and B), as well as strong apoptosis and caspase activation (Fig. 1, C-E), but surprisingly diminished expression of TAp73 (Fig. 1, A and B). Upon exposure to 50 μM cisplatin or 50 μg/ml 5-FU, TAp73 expression was initially increased up to 18 hr, but markedly declined afterwards, followed by enhanced expression of p53 target genes (Fig. 1B). Downregulation of TAp73 was not a consequence of caspase activation, as no TAp73 cleavage fragment was observed (Fig. S1, D and E), and caspase inhibition blocked apoptosis without affecting TAp73 depletion (Fig. S2). Furthermore, downregulation of TAp73 was also observed in *p53*-WT LOVO and RKO, and *p53*-mutant DLD1 colon cancer cells, as well as in mouse embryonic fibroblast (MEF) cells, following cisplatin treatment at a dose that induced substantial γH2Ax and apoptosis (Fig. 1F and data not shown). Therefore, TAp73 is induced by low levels of DNA damage, but is downregulated upon exposure to lethal doses of DNA damage.

### TAp73 inhibits apoptosis and expression of p53 target genes following excessive DNA damage

**Figure 2:**
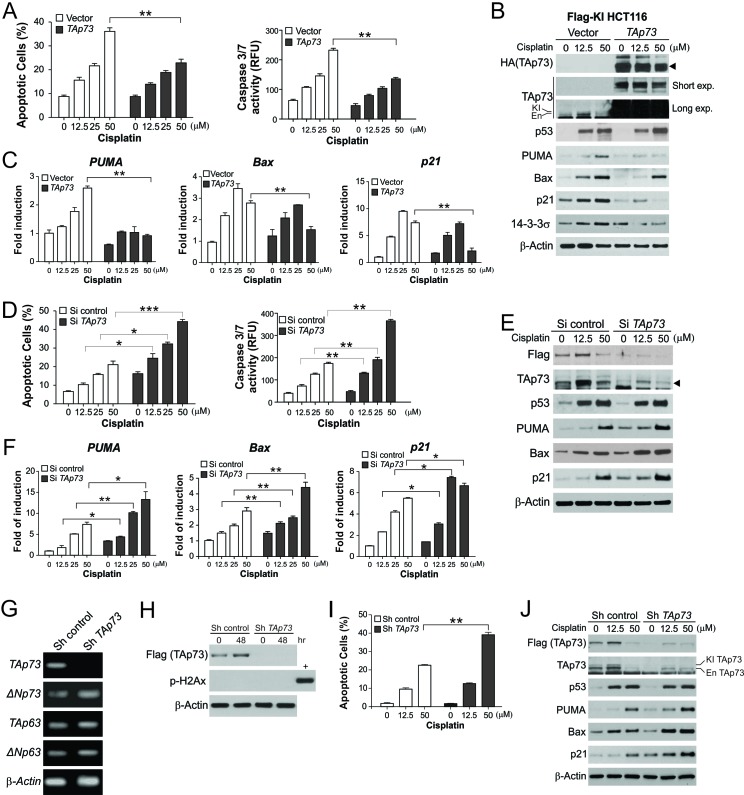
TAp73 suppresses apoptosis and the expression of p53 downstream target genes following extensive DNA damage **(A)** HCT116 cells with Flag-TAp73 KI were transfected with control or HA-TAp73α construct, and then treated with cisplatin at indicated concentrations for 24 hr. *Left*, analysis of apoptosis by nuclear staining; *Right*, analysis of caspase activity. **(B)** Following treatment as in (A), indicated proteins were analyzed by western blotting. TAp73 blots with short or long exposure (exp.) are presented to show transfected TAp73 and endogenous (En)/knock-in (KI) TAp73, respectively. **(C)** mRNA expression of *PUMA*, *Bax*, and *p21* in cells treated as in (A) was analyzed by real-time RT-PCR and normalized to the control *β-actin* and untreated samples. **(D)** HCT116 cells with Flag-TAp73 KI were transfected with control or *TAp73* siRNA, and treated with cisplatin at indicated concentrations for 24 hr. *Left*, analysis of apoptosis by nuclear staining; *Right*, analysis of caspase activity. **(E)** Following treatment as in (D), indicated proteins were analyzed by western blotting. **(F)** mRNA expression of *PUMA*, *Bax* and *p21* in cells treated as in (D) was analyzed by real-time RT-PCR. **(G)** HCT116 cells with stable transfection of *TAp73* or control shRNA were analyzed for the expression of *TAp73*, *ΔNp73*, *TAp63* and *ΔNp73* by RT-PCR. **(H)** HCT116 cells with stable transfection of *TAp73* or control shRNA at inoculation (0 hr) or after culturing for 48 hr were analyzed for TAp73 and γH2Ax by western blotting. Lysates from cisplatin-treated HCT116 cells were used as a positive control (+) for γH2Ax. **(I)** HCT116 cells with stable transfection of *TAp73* or control shRNA were treated with cisplatin at indicated concentrations for 24 hr. Apoptosis was analyzed by nuclear staining. **(J)** Following treatment as in (I), indicated proteins were analyzed by western blotting. Results in (A), (C), (D), (F) and (I) were expressed as means ± s.d. of three independent experiments. ***, *P* <0.001; **, *P* <0.01; *, *P* <0.05.

We then determined whether TAp73 downregulation is involved in initiating apoptosis in response to high levels of DNA damage. TAp73 transfection markedly suppressed apoptosis and caspase activation induced by 50 μM cisplatin, slightly reduced low levels of apoptosis and caspase activation induced by 12.5 μM cisplatin, but had no effect on untreated cells (Fig. 2A and S3A). Enforced TAp73 expression also inhibited protein and mRNA induction of p53 downstream targets, including *PUMA*, *Bax* and *p21*, in cells exposed to 12.5 or 50 μM cisplatin (Fig. 2, B and C). Conversely, knockdown of p73 by siRNA sensitized cells to apoptosis induction (Fig. 2D), and enhanced the protein and mRNA expression of *PUMA*, *Bax* and *p21* following cisplatin treatment at 12.5 or 50 μM (Fig. 2, E and F). These effects of TAp73 depletion were verified in HCT116 cells with stable knockdown of *TAp73* by shRNA, which by itself did not affect the expression of *ΔNp73* and *p63* isoforms, or induce genotoxic stress or apoptosis (Fig. 2, G-J; Fig. S3B). Modulating TAp73 expression also did not affect the induction of p53 by cisplatin (Fig. 2, B, E and J). Furthermore, TAp73 transfection or knockdown had similar effects on the induction of apoptosis and p53 target genes by cisplatin in RKO colon cancer cells (Fig. S4, A-D), as well as that by 5-FU in HCT116 cells (Fig. S5, A-D). In contrast, TAp73 transfection or knockdown did not affect p53-independent induction of apoptosis and PUMA by the kinase inhibitor staurosporine [17], although TAp73 was also downregulated in response to staurosporine treatment (Fig. S6, A-E).

**Figure 3:**
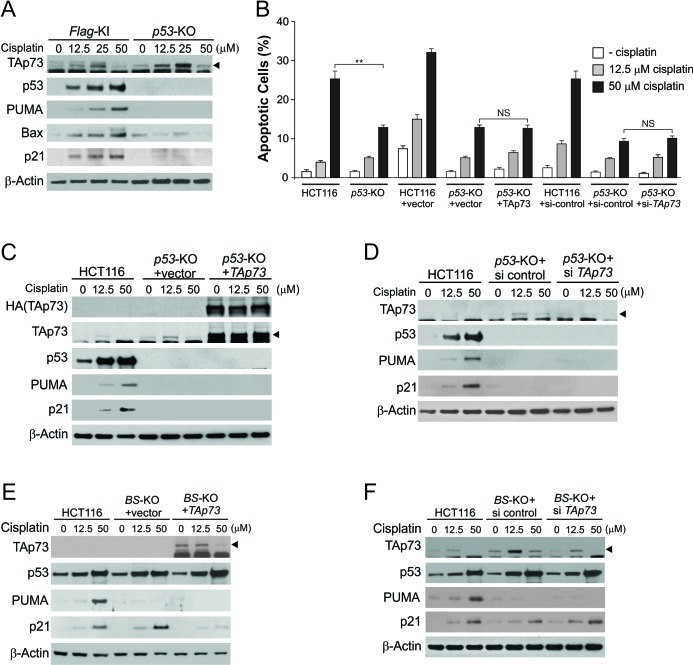
p53 is required for the effects of TAp73 on p53 target gene induction and apoptosis following extensive DNA damage **(A)** Flag-KI and *p53*-knockout (*p53*-KO) HCT116 cells were treated with cisplatin at indicated concentrations for 24 hr. Indicated proteins were analyzed by western blotting. **(B)** Apoptosis in cells treated as in (A), (C) and (D) was analyzed by nuclear staining. **, *P* <0.01; NS, not significant. **(C)** *p53*-KO HCT116 cells transfected with control or HA-TAp73α construct were treated with cisplatin at indicated concentrations for 24 hr. Indicated proteins were analyzed by western blotting. Lysates from HCT116 cells were loaded as a control. **(D)** *p53*-KO HCT116 cells transfected with control or *TAp73* siRNA were treated with cisplatin at indicated concentrations for 24 hr. Indicated proteins were analyzed by western blotting. Lysates from HCT116 cells were loaded as a control. **(E)** HCT116 cells with knockout of the p53 binding sites in the *PUMA* promoter (*BS*-KO) were transfected with control or HA-TAp73α expression construct, and then treated with cisplatin at indicated concentrations for 24 hr. Indicated proteins were analyzed by western blotting. **(F)** *BS*-KO cells were transfected with control or *TAp73* siRNA, and then treated with cisplatin at indicated concentrations for 24 hr. Indicated proteins were analyzed by western blotting.

To determine whether the effects of TAp73 are mediated through p53, we analyzed *p53*-knockout (*p53*-KO) HCT116 cells. Treating *p53*-KO cells with 50 μM cisplatin downregulated TAp73, but did not induce the expression of PUMA, Bax, and p21 (Fig. 3A), and led to a lower level of apoptosis compared to WT HCT116 cells (Fig. 3B). TAp73 transfection or knockdown did not affect the induction of apoptosis and p53 target genes in *p53*-KO cells (Fig. 3, B-D), and in *p53*-mutant DLD1 cells (Fig. S4, E and F). PUMA plays a critical role in DNA damage-induced and p53-dependent apoptosis [18]. To determine whether the effect of TAp73 on apoptosis is mediated through PUMA, we analyzed *PUMA*-knockout (*PUMA*-KO) HCT116 cells, which are less sensitive to DNA damage-induced apoptosis than WT cells [18]. TAp73 transfection or knockdown also did not substantially affect cisplatin-induced apoptosis without PUMA (Fig. S7, A-D). Both p53 and TAp73 can bind to the p53 responsive elements in the *PUMA* promoter upon DNA damage [19, 20]. To determine whether the effects of TAp73 on PUMA expression and apoptosis are mediated by its action on the *PUMA* promoter, we analyzed HCT116 cells with a deletion of the p53 binding sites in the *PUMA* promoter (*BS*-KO) [17]. In the absence of the p53 binding sites, TAp73 transfection or depletion did not impact cisplatin-induced apoptosis and PUMA expression, but still suppressed p21 expression as in WT HCT116 cells (Fig. 3, E and F; Fig. S7, E and F). These results demonstrate specific inhibitory effects of TAp73 on DNA damage-induced apoptosis, which are meditated by p53 through its downstream target genes.

### TAp73 inhibits DNA binding and transactivation by p53 following DNA damage

**Figure 4:**
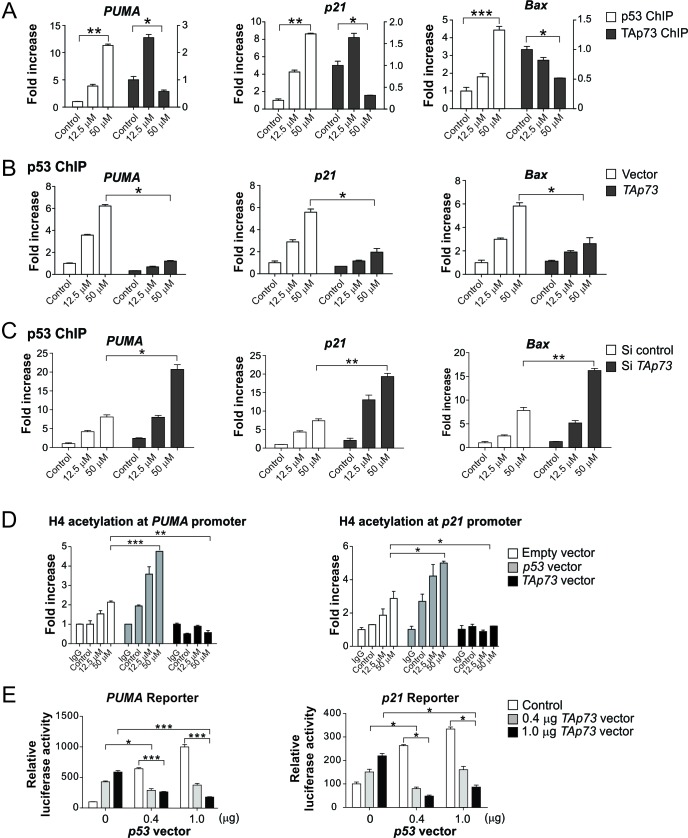
TAp73 inhibits p53 binding to its target gene promoters following excessive DNA damage **(A)** HCT116 cells with Flag-TAp73 KI were treated with cisplatin at indicated concentrations for 24 hr. Chromatin immunoprecipitation (ChIP) followed by quantitative real-time PCR was used to analyze the binding of p53 and TAp73 to the promoters of *PUMA*, *Bax* and *p21*, using anti-p53 antibody and anti-Flag-conjugated beads, respectively. Results were normalized to those of IgG (for p53 ChIP) and parental HCT116 (for TAp73 ChIP), which were used as negative controls for ChIP, and plotted as fold of enrichment relative to the control. **(B)** Flag-KI HCT116 cells were transfected with control or HA-TAp73α construct, and then treated with cisplatin at indicated concentrations for 24 hr. Occupancy of p53 at the indicated promoters was analyzed by ChIP as in (A). **(C)** Flag-KI HCT116 cells were transfected with control or *TAp73* siRNA, and then treated with cisplatin at indicated concentrations for 24 hr. Binding of p53 to the indicated promoters was analyzed as in (A). **(D)** HCT116 cells were transfected with p53 or TAp73α, and treated with cisplatin at indicated concentrations for 24 hr. Histone H4 acetylation, a marker of transcriptional activation, in *PUMA* and *p21* promoters was analyzed by ChIP using an antibody specific for acetylated H4. The results were normalized to those of input. **(E)** *p53*-KO HCT116 cells were co-transfected with a PUMA or p21 luciferase reporter, along with indicated amounts of p53 and TAp73α expression vectors, or control empty vectors. Activation of the *PUMA* and *p21* reporters was measured 24 hr after transfection, as described in the Materials and Methods. Results in (A)-(E) were expressed as means ± s.d. of three independent experiments. ***, *P* <0.001; **, *P* <0.01; *, *P* <0.05.

To determine a potential role of p53 and TAp73 occupancy on promoters of p53 target genes, we used chromatin immunoprecipitation (ChIP) to analyze the binding of p53 and TAp73 to several promoters in cisplatin-treated Flag-KI cells. In response to 12.5 μM cisplatin for 24 hr, p53 was recruited to its target gene promoters, while TAp73 was selectively recruited to the promoters of *PUMA*, *p21* and *14-3-3σ* (Fig. 4A and S8A). In response to 50 μM cisplatin for 24 hr, the binding of p53 to its target promoters markedly increased, while that of TAp73 decreased (Fig. 4A and S8A). The binding of p53 to its target gene promoters upon cisplatin exposure was markedly suppressed by transfection with TAp73 (Fig. 4B and S8B), while significantly increased upon knockdown of *TAp73* (Fig. 4C and S8C). Furthermore, cisplatin dose escalation resulted in increased Histone H4 acetylation, a chromatin remodeling marker of transcriptional activation, in the *PUMA* and *p21* promoter regions (Fig. 4D). TAp73 transfection almost completely abolished cisplatin-induced H4 acetylation, whereas p53 transfection had an opposite effect (Fig. 4D). TAp73 transfection also suppressed the activation of *PUMA* and *p21* luciferase reporters by p53 in a dose-dependent manner in *p53*-KO cells (Fig. 4E). Together, these results indicate that upon sub-lethal DNA damage, TAp73 restricts p53 transcriptional activity, while excessive DNA damage downregulates TAp73 to unleash the full activity of p53 to drive apoptosis induction.

### TAp73 and p53 inhibit each other in DNA binding and form protein complexes

**Figure 5:**
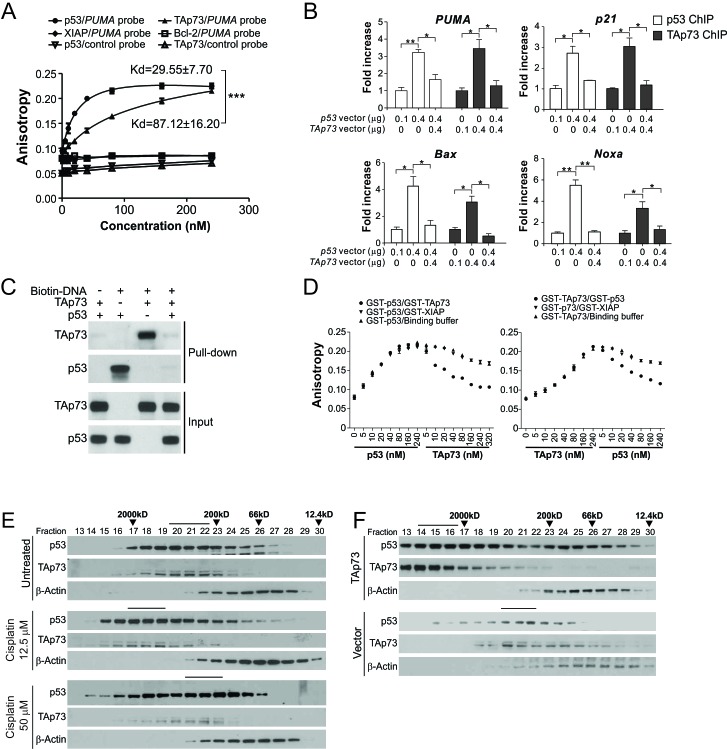
TAp73 inhibits the DNA binding of p53 through complex formation, rather than promoter competition **(A)** Fluorescence polarization analysis of DNA binding by p53 and TAp73. Fluorescence polarization was performed by titrating GST-TAp73α and GST-p53 into 4 nM FAM-labeled *PUMA* promoter oligonucleotide. GST-XIAP, GST-Bcl-2 and a non-specific DNA sequence (NS) were used as negative control. Results were expressed as means ± s.d. of three independent experiments. ***, *P*<0.001. **(B)** *p53-*KO HCT116 cells were transfected with p53, HA-TAp73α, or both proteins at indicated amounts. Binding of p53 and TAp73 to the promoters of *PUMA*, *p21*, *Bax* and *Noxa* was analyzed by ChIP using anti-p53 antibody and anti-HA, respectively, followed by quantitative real-time PCR. Results were normalized to those of IgG, which was used as a negative control, and expressed as means ± s.d. of three independent experiments. **, *P* <0.01; *, *P* <0.05. **(C)** Far-western blotting analysis of p53 and TAp73 binding to the *PUMA* promoter. Biotinylated *PUMA* promoter probe was incubated with cell lysates from *p53-*KO HCT116 cells transfected with TAp73α, p53, or both, followed by pull-down with streptavidin-conjugated beads and analysis of p53 and TAp73 by western blotting. **(D)** Fluorescence polarization analysis of the interference of p53 and TAp73 on each other's DNA binding. *Left*, p53 was titrated into 4 nM of FAM-labeled PUMA promoter oligonucleotide to saturation, followed by the addition of GST-TAp73 for titration. *Right*, TAp73 titration followed by p53 titration. GST-XIAP or blank binding buffer was used as negative control. **(E)**Whole cell extracts (~3 mg) were prepared from HCT116 cells treated with 0, 12.5 or 50 μM cisplatin, and then subjected to gel filtration column chromatography. An equal volume of eluted proteins in each fraction was analyzed by immunoblotting with the indicated antibodies. Molecular weight markers and three peak fractions for each sample are indicated. **(F)** HCT116 cells transfected with control or HA-TAp73α vector were analyzed as in (E).

Several possibilities may explain how TAp73 inhibits p53-mediated transactivation, such as competitive binding to the same promoter and inhibiting formation of transcription-competent p53 tetramers. We examined the former by an equilibrium binding assay using fluorescence polarization and GST-tagged recombinant p53 and TAp73 proteins. Recombinant p53 and TAp73 were found to bind to a synthetic *PUMA* promoter probe with an equilibrium binding constant (Kd) of 29.55 nM and 87.12 nM, respectively (Fig. 5A), suggesting that p73 is less efficient in DNA binding compared to p53 and may not directly compete out p53 at the p53 responsive elements. ChIP analysis showed that co-transfection of p53 and TAp73 attenuated the binding of both proteins to the promoters of *PUMA*, *p21*, *Bax* and *Noxa*, relative to transfection of p53 or TAp73 alone (Fig. 5B). Far-western blotting showed that co-expression of p53 and TAp73 in *p53*-KO cells abolished the *in vitro* binding of both proteins to the *PUMA* promoter probe (Fig. 5C). Furthermore, the binding of p53 or TAp73 to the *PUMA* promoter probe was inhibited by titration of TAp73 or p53 in fluorescence polarization assays, whereas the control GST-tagged X-linked IAP (XIAP) had no effect on the binding of p53 and TAp73 (Fig. 5D). Therefore, the presence of TAp73 can impair the ability of p53 to bind to DNA. Using gel filtration column chromatography, we found that both p53 and TAp73 peak fractions concordantly shifted to a higher molecular weight (MW) range in cells treated with 12.5 μM cisplatin (Fig. 5E), compared to untreated cells, suggesting formation of large and likely transcriptionally incompetent complexes. In contrast, the shifts of p53 and TAp73 fractions were less pronounced in cells treated with 50 μM cisplatin (Fig. 5E). TAp73 transfection led to similar shifts of p53 peak fractions as that caused by 12.5 μM cisplatin (Fig. 5F). Together, these results suggest that TAp73 inhibits p53 activity by forming protein complexes that are unable to efficiently bind to DNA.

### Downregulation of p73 by ubiquitin/proteasome-mediated E2F1 degradation in response to excessive DNA damage

**Figure 6:**
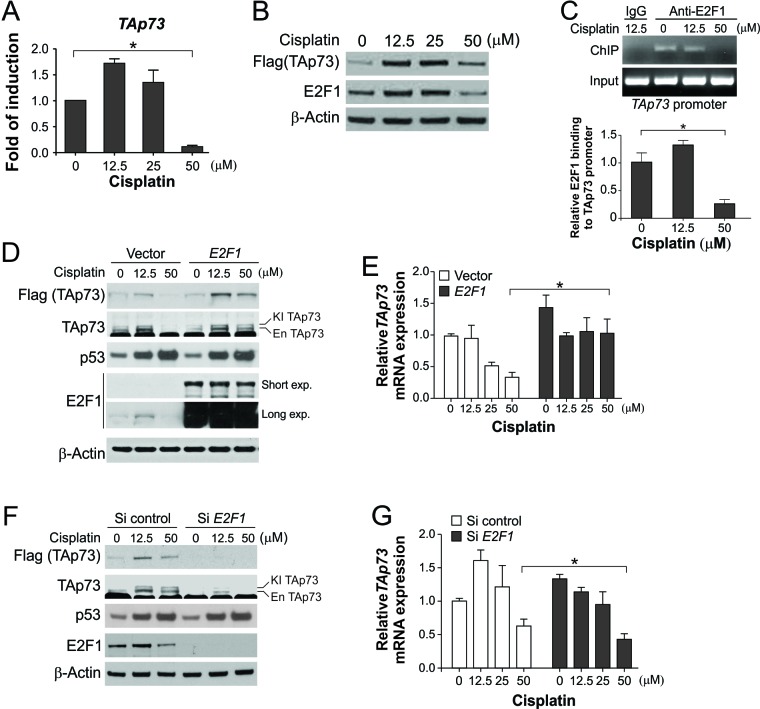
Downregulation of E2F1-mediated *TAp73* transcription following extensive DNA damage **(A)** HCT116 cells were treated with cisplatin at indicated concentrations for 24 hr. *TAp73* mRNA expression was analyzed by real-time RT-PCR. **(B)** Following treatment of HCT116 cells as in (A), indicated proteins were analyzed by western blotting. **(C)** HCT116 cells were treated with cisplatin at indicated concentrations for 24 hr. ChIP was performed using anti-E2F1 antibody, followed by PCR analysis using primers flanking the genomic region that contains the E2F1 binding sites in the *TAp73* promoter. IgG was used as a negative control. *Upper,* analysis of PCR products by gel electrophoresis; *Lower*, quantification of the ChIP results by real-time PCR. **(D)** HCT116 cells were transfected with control or HA-E2F1 expression construct, and then treated with cisplatin at indicated concentrations for 24 hr. Indicated proteins were analyzed by western blotting. **(E)** Analysis of *TAp73* mRNA expression in cells treated as in (D) by real-time RT-PCR. **(F)** HCT116 cells were transfected with control or *E2F1* siRNA, and then treated with cisplatin at indicated concentrations for 24 hr. Indicated proteins were analyzed by western blotting. **(G)** Analysis of *TAp73* mRNA expression in cells treated as in (F) by real-time RT-PCR. RT-PCR results in (A), (E) and (G) were normalized to the control *β-actin* and expressed as means ± s.d. of three independent experiments. *, *P* <0.05.

We next investigated the mechanism by which TAp73 expression is regulated in response to different levels of DNA damage. The mRNA expression of *TAp73*, but not that of *ΔNp73, TAp63,* and *ΔNp63*, was markedly decreased following treatment with 50 μM cisplatin (Fig. 6A and S9). The expression of E2F1, a key transcription factor that regulates TAp73 and DNA damage response [21, 22], as well as E2F1 bound to *p73* promoter, were decreased upon 50 μM cisplatin (Fig. 6, B and C). E2F1 depletion was also detected in cells treated with 50 μg/ml 5-FU (Fig. S10A). E2F1 transfection blocked the downregulation of TAp73 protein and mRNA in cells treated with 50 μM cisplatin (Fig. 6, D and E). Conversely, *E2F1* knockdown enhanced TAp73 depletion (Fig. 6, F and G). Similar effects of E2F1 on TAp73 expression were also observed in RKO colon cancer cells exposed to cisplatin (Fig. S10, B and C).

**Figure 7:**
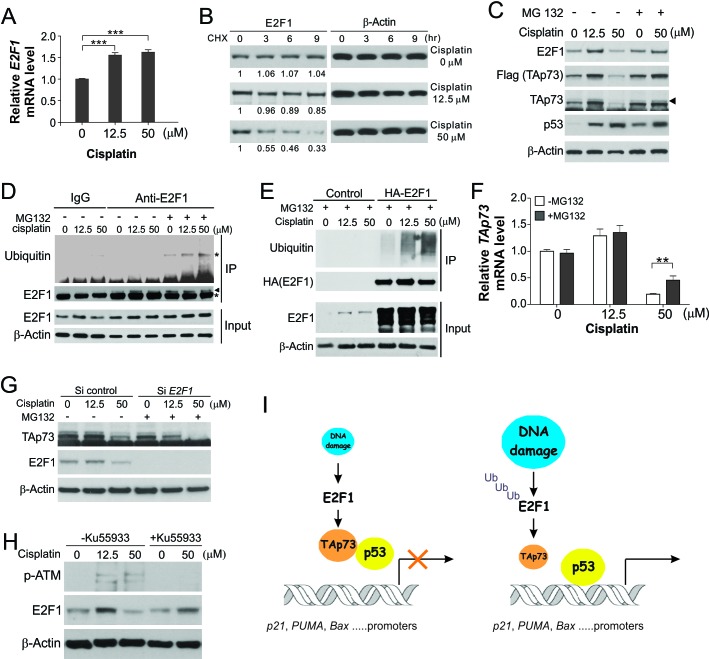
E2F1 is degraded through the ubiquitin/proteasome pathway in response to excessive DNA damage **(A)** HCT116 cells were treated with cisplatin at indicated concentrations for 18 hr. *E2F1* mRNA expression was analyzed by real-time RT-PCR. Results were normalized to the control β-actin. ***, P <0.001. **(B)** HCT116 cells were treated with cisplatin at indicated concentrations along with 100 μM of the protein synthesis inhibitor cycloheximide (CHX). E2F1 and β-actin expression at indicated time points was analyzed by western blotting. Relative E2F1 expression of each sample, normalized to that of the loading control β-actin, is indicated, with that of the untreated cells arbitrarily set as 1.0. **(C)** HCT116 cells with or without cisplatin treatment at indicated concentrations for 18 hr were exposed to 5 μM of MG132 or the control DMSO for additional 6 hr. Western blotting was used to analyze indicated proteins. **(D)** HCT116 cells treated with cisplatin and/or MG132 as in (C) were analyzed by immunoprecipitation using anti-E2F1 antibody, followed by western blot analysis with anti-ubiquitin antibody. The IP inputs were normalized based on E2F1 expression. Arrowhead indicates E2F1 after pull-down, and asterisks indicate non-specific bands. **(E)** HCT116 cells were transfected with HA-E2F1, and then treated with cisplatin and/or MG132 as in (C). Immunoprecipitation was performed using anti-HA antibody, followed by western blot analysis with anti-ubiquitin antibody. **(F)** *TAp73* mRNA expression in HCT116 cells treated as in (C) was analyzed by real-time RT-PCR. Results were normalized to the control *β-actin*. **, *P* <0.01. **(G)** HCT116 cells were transfected with control or *E2F1* siRNA, treated with cisplatin and/or MG132 as in (C). Indicated proteins were analyzed by western blotting. **(H)** HCT116 cells were treated with cisplatin at indicated concentrations, along with 10 μM of the ATM inhibitor Ku55933 for 24 hr. Western blotting was used to analyze phospho-ATM (p-ATM; Ser1981) and E2F1. **(I)** A model of how p73 affects p53 activity in response to different levels of DNA damage.

We further investigated the mechanism of E2F1 depletion. Unlike *TAp73*, the mRNA expression of *E2F1* was not decreased, but slightly increased upon cisplatin exposure, compared to untreated cells (Fig. 7A), suggesting a post-transcriptional mechanism of E2F1 depletion upon 50 μM cisplatin. The increased *E2F1* mRNA expression may account for the increased E2F1 protein expression upon 12.5 μM cisplatin (Fig. 6B). We then tested whether ubiquitin/proteasome-mediated protein degradation is involved in E2F1 depletion. The half-life of E2F1 was decreased from >9 hr in response to 12.5 μM cisplatin to 3–6 hr upon 50 μM cisplatin (Fig. 7B). Proteasome inhibitor MG132 treatment reverted the declined E2F1 expression in response to 50 μM of cisplatin (Fig. 7C). Ubiquitination of both endogenous and transfected E2F1 (analyzed in normalized cell lysates) was markedly enhanced following treatment with 50 μM cisplatin, compared to untreated cells or those treated with 12.5 μM cisplatin (Fig. 7, D and E). MG132 treatment also impeded the declined expression of *p73* mRNA and protein (Fig. 7, C and F). Knockdown of E2F1 suppressed the effect of MG132 on TAp73 expression in response to 50 μM cisplatin (Fig. 7G). Since MG132 alone only slightly increased TAp73 level (Fig. 7C), the E2F1-dependent effect of MG132 on TAp73 expression suggests that downregulation of TAp73 is mediated by proteasomal degradation of E2F rather than TAp73 itself. However, the effects of MG132 cannot be directly correlated with the E2F1/p73 pathway, as proteasome inhibition has multiple cellular effects including severe cellular damage. To further understand the mechanism of E2F1 degradation, we probed ATM, an upstream regulator of DNA damage response [4]. The E2F1 depletion was found to be mediated by ATM, as co-treatment with the ATM inhibitor Ku55933 restored E2F1 expression upon 50 μM cisplatin (Fig. 7H). Together, these results demonstrate that lethal DNA damage triggers ubiquitin/proteasome-mediated E2F1 degradation and p73 downregulation to facilitate p53-dependent apoptosis.

### MATERIALS AND METHODS

#### Cell culture and drug treatment

Human colorectal cancer cell lines, including HCT116, RKO, LOVO, which contain WT p53, and DLD1, which contains mutant p53, were obtained from American Type Culture Collection (ATCC; Manassas, VA). *p53*-KO and *PUMA*-KO HCT116 cells were obtained from Dr. Bert Vogelstein (Sidney Kimmel Comprehensive Cancer Center at Johns Hopkins). p53-binding site knockout (*BS*-KO) HCT116 cells and immortalized MEF cells were previously described [17]. Colon cancer cells were maintained in McCoy's 5A medium (Invitrogen). MEF cells were cultured using Dulbecco's modified Eagle's medium (BioWhittaker). All cell culture media were supplemented with 10% fetal bovine serum (HyClone), 100 U/ml of penicillin, and 100 µg/ml of streptomycin. Cells were maintained at 37 ^o^C in 5 % CO_2_ atmosphere.

Cells were plated in 12-well plates at 40–50% density 24 hr before treatment. Cisplatin, etoposide, 5-Fluorouracil (5-FU), camptothecin and staurosporine (Sigma) were diluted to different concentrations in cell culture medium. In some experiments, cells were treated with DNA damaging drugs for 18 hr, and then with the proteasome inhibitor MG 132 (Sigma) for an additional 6 hr.

#### Knock-in of a triple-Flag tag in TAp73

Vectors for knocking-in Flag-tagged TAp73 were constructed using the pUSER-rAAV (recombinant adeno-associated virus) System [23]. Briefly, two homologous arms of ~1.32 kb and 0.995 kb, respectively, flanking the second intron of *TAp73* were inserted between 2 USER sites in the AAV shuttle vector pTK-Neo-USER (Fig. S1A). Coding sequence of a triple-Flag tag was introduced by PCR into the left arm immediately 3' following the initiation ATG codon of TAp73α, as previously described [24]. For gene targeting, HCT116 cells, in which TAp73 is the most prominently expressed p73 isoform [25], were infected with the targeting rAAV and selected by G418 (0.5 mg/mL; Mediatech) for 3 weeks. G418-resistant clones were pooled and screened by PCR for targeting events using the primer pair listed in Table S1. After gene targeting, *Neo* was excised by Cre adenovirus infection. Gene targeting was verified by sequencing of genomic DNA and analysis of Flag-TAp73 expression using western blotting.

#### Apoptosis and caspase activity assays

After treatment, adherent and floating cells were harvested and resuspended with PBS solution containing 3.7 % formaldehyde, 0.5 % Nonidet P-40, and 10 μg/ml Hoechst 33258 (Invitrogen). Apoptosis was assessed through microscopic visualization and counting of cells with condensed chromatin and micronucleations as described [26]. For each measurement, at least three independent experiments and a minimum of 300 cells were analyzed. For annexin V/propidium iodide (PI) staining, cells were stained by annexin Alexa-488/PI (Invitrogen) following the manufacturer's instruction, and then analyzed by flow cytometry. Caspase activity was measured using the SensoLyte Homogeneous AMC Caspase-3/7 Assay Kit (Anaspec). Briefly, 1.0×10^4^ HCT116 cells were seeded in each well of a 96-well plate. After treatment, cells were incubated with the caspase substrate Ac-DEVD-AMC at room temperature for 40 min. Fluorescence was measured using a Wallac Victor 1420 Multilabel Counter (PerkinElmer), and the data are presented as relative fluorescence units.

#### Transfection and siRNA/shRNA knockdown

Transfection of expression constructs and small interfering RNA (siRNA) was performed using Lipofectamine 2000 (Invitrogen) according to the manufacturer's instructions. Expression constructs, including pCMV-Neo-Bam p53 WT (from Dr. Bert Vogelstein), HA-TAp73α-pcDNA3 (from Dr. William Kaelin) and pSG5L-HA-E2F1 (from Dr. William Sellers), were obtained from Addgene (Cambridge, MA). siRNA (Dharmacon) of 200 pmol each was transfected into cells 24 hr before drug treatment. The siRNA sequences have been previously described, including those for human *TAp73* (5'-GGATTCCAGCATGGACGTCTT-3') [27], and human *E2F1* (5'-GGACCTTCGTAGCATTGCA-3') [28]. Stable *TAp73* knockdown cells were generated by transducing HCT116 cells with the pLKO.1-puro lentiviral vector (Addgene) expressing shRNA with the same targeting sequence as the *TAp73* siRNA. Lentiviral particles were generated by co-transfecting 293T cells with the lentiviral vector, pMD2.G (VSVG), pMDLg/pRRE, and pRSV-REV (Addgene). Following lentiviral transduction, cells were plated in 96-well plates in the presence of puromycin (2 μg/ml; EMD/Millipore). TAp73 expression of the puromycin-resistant clones was then analyzed by Western blotting.

#### Chromatin immunoprecipitation (ChIP)

For analysis of promoter binding by p53 and E2F1, and histone H4 acetylation, ChIP was carried out by using the Chromatin Immunoprecipitation Assay Kit (Upstate/Millipore), as previously described [17]. Antibodies against p53 (SC-126), E2F1 (SC-251) (Santa Cruz Biotechnology), acetylated histone H4 (#06–598; Upstate/Millipore) were used. Isotype-matched IgG (R&D System) were used as control for IP. For analysis of promoter binding by TAp73, HCT116 cells with knock-in of Flag-TAp73 were treated and analyzed. IP was performed using EZview Red ANTI-FLAG M2 Affinity Gel (Sigma) according to manufacturer's instructions. Parental HCT116 cells were used as the negative control. After ChIP, the IP complex was analyzed by PCR using the primers listed in Table S1. In some experiments, cells were first transfected with HA-TAp73α, *TAp73* siRNA, p53, or both HA-TAp73α and p53. Twenty-four hr after transfection, cells were treated with cisplatin and analyzed by ChIP for p53 using anti-p53, or for TAp73 using anti-HA antibody (Santa Cruz Biotechnology).

#### Luciferase reporter assay

For analysis of *PUMA* and *p21* promoter activities, *p53*-KO HCT116 cells were transfected with a *PUMA* luciferase reporter containing the p53 binding site (Fragment A) [29] or *p21* luciferase reporter (Addgene, Plasmid 16451), along with HA-p73α-pcDNA3 and/or pCMV-Neo-Bam p53 WT, and the control β-galactosidase reporter pCMVβ (Promega, Madison, WI, USA). Luciferase activities were measured and normalized to those of β-galactosidase, as previously described [30]. Experiments were performed in triplicate and repeated at least three times.

#### Western blot and antibodies

Cells were lysed with 2×Laemmli sample buffer and subjected to western blotting, as previously described [31]. Antibodies used for western blotting included those against p73 (A300–136A; Bethyl Labs), PUMA [31], p53 (SC-126), HA (SC-805), Bax (SC-493), p21 (SC-397), 14-3-3σ (SC-100638) and E2F1 (SC-251) (Santa Cruz Biotechnology), Flag (F3165), β-Actin (A5441) (Sigma), phospho-ATM (Ser1981) (ab81292; Abcam) and ubiquitin (#550944; BD Transduction).

#### Real-time reverse transcriptase (RT) PCR

Total RNA was isolated by using Quick-RNA mini-prep kit (ZYMO Research, Irvine, CA) according to the manufacturer's instructions, and subjected to reverse transcription to generate cDNA. Real-time RT-PCR was performed on a CFX96 Real-Time PCR system (Bio-Rad, Hercules, CA) with SYBR Green (Invitrogen). Primers for RT-PCR are listed in Table S1. Agarose gel electrophoresis of the PCR products was used to verify the specificity of PCR amplification.

#### Ubiquitination Assay

To detect E2F1 ubiquitination following cisplatin treatment, HCT116 cells with or without transfection of pSG5L-HA-E2F1 were treated with cisplatin for 18 hr, and then with 5 μM MG 132 for an additional 6 hr. IP of endogenous and transfected E2F1 was performed using anti-E2F1 and anti-HA, respectively. IP complexes were pulled down using EZview Red Protein G Affinity Gel (Sigma) according to the manufacturer's instructions, lysed in 2×Laemmli sample buffer, and analyzed for ubiquitin by western blotting.

#### Analysis of DNA binding by p53 and TAp73

To analyze DNA binding by p53 and p73 using fluorescence anisotropy/polarization, aliquots of stock solutions of GST-p53 (2.5 μM) or GST-TAp73α (2.0 μM) (SignalChem) were added to HPLC-purified 6-carboxyfluorescein (6-FAM)-labeled DNA duplex (5'-/56-FAM/ CGCGCCTGCAAGTCCTGACTTGTCCGCGGC-3'; Integrated DNA Technologies) containing the p53 binding sites in the *PUMA* promoter at 4 nM in a 500-μL reaction volume at room temperature in a buffer containing 100 mM NaCl, 10 mM tris-HCl, PH7.5, 10% (vol/vol) glycerol, 5 mM DTT, and 0.2 mg/mL bovine serum albumin. After mixing by stirring for 10 sec and incubation for 30 sec, fluorescence anisotropy was recorded on a Varian Cary Eclipse Fluorescence Spectrophotometer (Agilent Technologies), with λex set at 480 nm and λem at 530 nm. A 6-FAM-labeled 26-bp random DNA duplex (5^'^-/56-FAM/AATGGAAATTTCCGGAAATTTCCATT-3') was used as negative control with an initial concentration of 4 nM in 500-μL reaction volume as well. Data were analyzed as previous described [32].

To analyze DNA binding by p53 and TAp73 using far-western blotting, cell lysates prepared from *p53*-KO HCT116 cells transfected with p53, HA-TAp73α, or both proteins were incubated with 2 μg of the duplex *PUMA* promoter probe with or without 5' biotin label for 90 min at room temperature, and then with streptavidin-conjugated magnetic beads (Pierce) at 4^o^C for 2 hr. After isolation by centrifugation and two washes, the beads were boiled in 2×Laemmli sample buffer and subjected to western blotting for the transfected p53 and TAp73.

#### Gel filtration column chromatography

Gel filtration column chromatography was carried out as previously described [33]. In brief, 3 mg of whole cell extracts prepared in a column elution buffer (30 mM HEPES, pH 7.4; 150 mM NaCl; 10% glycerol; 0.1% NP-40; 5 μg/ml DNase I; and protease inhibitor cocktail [Roche]) were loaded on a column packed with Superose 6 prep grade gel (GE Healthcare), and 500-μl elution was collected in each fraction. Equal volumes of eluted fractions were subjected to western blotting. A mixture of protein markers containing blue dextran (2,000 kDa), β-amylase (200 kDa), bovine serum albumin (66 kDa), and cytochrome *c* (12.4 kDa) was used as the molecular weight standard.

### DISCUSSION

In response to mild and repairable DNA damage, low levels of p53 induction tend to favor growth arrest, whereas severe, extended, or irrepairable DNA damage often leads to apoptosis. Our results indicate that TAp73 plays a critical role in controlling overall p53 activity and cell fate determination in response to DNA damage (Fig. 7I). TAp73 binds to p53 and inhibits p53-mediated transactivation following mild DNA damage, resulting in limited activation of p53 target genes to favor growth inhibition. Excessive DNA lesions lead to TAp73 depletion and enhanced p53 activation and trigger apoptosis. Although the expression of p53 target genes, such as *PUMA* and *p21*, is similarly modulated in response to different levels of DNA damage through TAp73 and p53, the impact of these changes on cell fates may be different due to differential threshold activities involved in different biological responses. While modest induction of p21 is probably sufficient to halt cell cycle progression, strong induction of PUMA and other apoptosis regulators may be necessary for efficient apoptosis induction. Following high levels of DNA damage, TAp73 expression sharply declined (Fig. 1A and B), but apoptosis induction was less dramatic and exhibited a dose response (Fig. 1C). This can probably be explained by the indirect and partial effects of TAp73 on apoptosis induction, which is regulated by TAp73 through p53 and its target genes, as well as other apoptosis regulators.

Differential p53 response can result from selective expression of p53 targets through several mechanisms. Variations in binding affinities of p53 for its target promoters can profoundly affect promoter choice and cell fate determination [34]. A number of p53 partner proteins have been implicated in modulating the selection of p53 targets, such as Brn3a and YB1, which favor growth arrest, and ASPP family members, p300, and CAS, which are proapoptotic [35, 36]. Hzf, a zinc finger protein that interacts with p53, can selectively induce p53 targets involved in growth arrest. Prolonged DNA damage exposure led to Hzf degradation and enhanced expression of the proapoptotic targets of p53 [37]. TAp73 appears to be different from Hzf as it regulates overall p53 activity rather than target selectivity. While TAp73 is modulated in response to altered extent of DNA damage, Hzf is altered upon changes in duration of DNA damage. Furthermore, different post-translational modifications of p53 also regulate selective expression of p53 targets [38]. In addition to p53, excessive DNA damage can also activate an NF-kB-dependent pathway to induce cytokine secretion and apoptosis [16]. Therefore, a set of well-orchestrated events including TAp73 depletion are set forth in response to lethal DNA damage to signal cells death through apoptosis.

TAp73 contains a highly-conserved DNA-binding domain and shares sequence specificity with p53 [39]. It is possible that TAp73 simply competes with p53 for DNA binding to prevent the activation of p53 target genes [40]. However, our data are inconsistent with this hypothesis. TAp73 binds less tightly than p53 to the *PUMA* promoter (Fig. 5A). TAp73 and p53 co-expression or co-incubation abolished DNA binding by both proteins in cells and *in vitro* binding assays (Fig. 5, B-D). Several p73 and p63 isoforms can physically interact with each other or with mutant p53 [41, 42]. In the context of mild DNA damage, p73 seems to antagonize p53 activity through the formation of large protein complexes (Fig. 5, E and F). These complexes might lack symmetric conformation and cooperativity required for binding to the p53 responsive elements. In support of our findings, DNA binding cooperativity of p53 has been shown to determine the extent of DNA damage-induced apoptosis [43]. It should be noted that the DNA binding and transactivation of both p53 and TAp73 are subjected to extensive modifications following DNA damage [44]. Our data cannot completely rule out the possibility that p53 and TAp73 compete with each other for DNA binding.

Despite numerous studies on p53 homologues and clarification of TAp73 as a tumor suppressor [9, 10], the precise function of TAp73 in DNA damage response and tumor suppression is not fully understood [45]. TAp73 is often proapoptotic, but could be antiapoptotic under certain circumstances [46, 47]. In contrast to our findings, TAp73 was shown to be proapoptotic in HCT116 cells in response to the MDM2 inhibitor Nutlin-3, which might be due to the effects of Nutlin-3 on p53 and other MDM2 targets [48]. The proapoptotic activity of TAp73 is regulated by DNA damage-dependent acetylation [49] and cellular localization [50]. Our results show for the first time that the interplay between TAp73 and p53 is dependent on extent of DNA damage and TAp73 levels. TAp73 can be antiapoptotic by antagonizing p53 when genotoxic stress is not severe enough to pose danger to an organism. However, TAp73 accumulation without concurrent p53 stabilization or in a p53-deficient background may allow TAp73 to induce p53 target genes and cell death [51], even though it is a less effective transactivator than p53. TAp73 also exhibits promoter selectivity and has a number of unique target genes that are distinct from those of p53 [52]. Antagonizing p53 by TAp73 in DNA damage response may also explain why TAp73 is not a classical tumor suppressor. TAp73 is rarely mutated in human tumors and even overexpressed in some tumors [45]. p73-deficient mice, including *TAp73*-knockout mice, are different from p53-null mice in both animal development and tumor incidence [9, 12, 53]. TAp73 was previously found to interfere with p53 in regulating telomerase activity [54]. In line with its pro-survival function, TAp73 has recently been shown to promote cell growth by activating AP-1 target genes [55], or through the pentose phosphate pathway [56]. It is likely that other p53 homologues, such as TAp73β, ΔNp73, and p63 isoforms, may help to further fine tune the activities of p53 and p73 in response to different stress conditions [57].

TAp73 expression is mainly regulated at the transcriptional level by E2F1 [22]. Upon sub-lethal DNA damage, E2F1 induction sustains TAp73 expression to keep the activity of p53 in check (Fig. 7I). Extensive DNA lesions trigger ubiquitin/proteasome-mediated E2F1 degradation (Fig. 7I), allowing p53 to fully activate its target genes and induce apoptosis. Similar to TAp73, E2F1 has been shown to promote apoptosis in a p53-deficient background [58]. The activity of TAp73 can also be regulated by a number of other proteins. For example, TAp73 can be phosphorylated by the tyrosine kinase c-Abl during apoptosis induced by cisplatin and other DNA damaging agents [59, 60]. TAp73 expression can also be regulated by p53 in response to oxidative stress [61]. A remaining question is how E2F1 degradation is triggered in response to lethal DNA damage, and our data suggest the involvement of ATM (Fig. 7H), which initiates a series of downstream signaling events following excessive DNA lesions [16]. E2F1 can also be methylated at lysine 185 by Set9 methyltransferase, which prevents DNA damage-induced E2F1 accumulation and p73 activation [28].

TAp73 and other p53 homologues can be activated by chemotherapeutic drugs and γ-irradiation, and implicated as a major determinant of chemotherapy sensitivity, particularly in p53-deficient tumors [62]. Our data suggest that the influence of TAp73 on p53 in both cancer and normal cells needs to be taken into consideration in the use of TAp73 as a therapeutic target. In conclusion, our study has uncovered that TAp73 helps to antagonize the action of p53. In response to high levels of DNA damage, the depletion of TAp73 through proteasomal degradation of E2F1 is a prerequisite to trigger p53-dependent apoptosis to eliminate cells harboring extensive genomic damage.

## SUPPLEMENTARY FIGURES AND TABLE


