# Supplementary material for: TAp73 promotes cell survival upon genotoxic stress by inhibiting p53 activity
Source: Oncotarget. 2014 Sep 22;5(18):8107–22. doi: 10.18632/oncotarget.2440 (PMC4226670; doi:10.18632/oncotarget.2440)
Supplement: Supplementary file 1 [file oncotarget-05-8107-s001.pdf]

SUPPLEMENTARY FIGURES AND TABLE

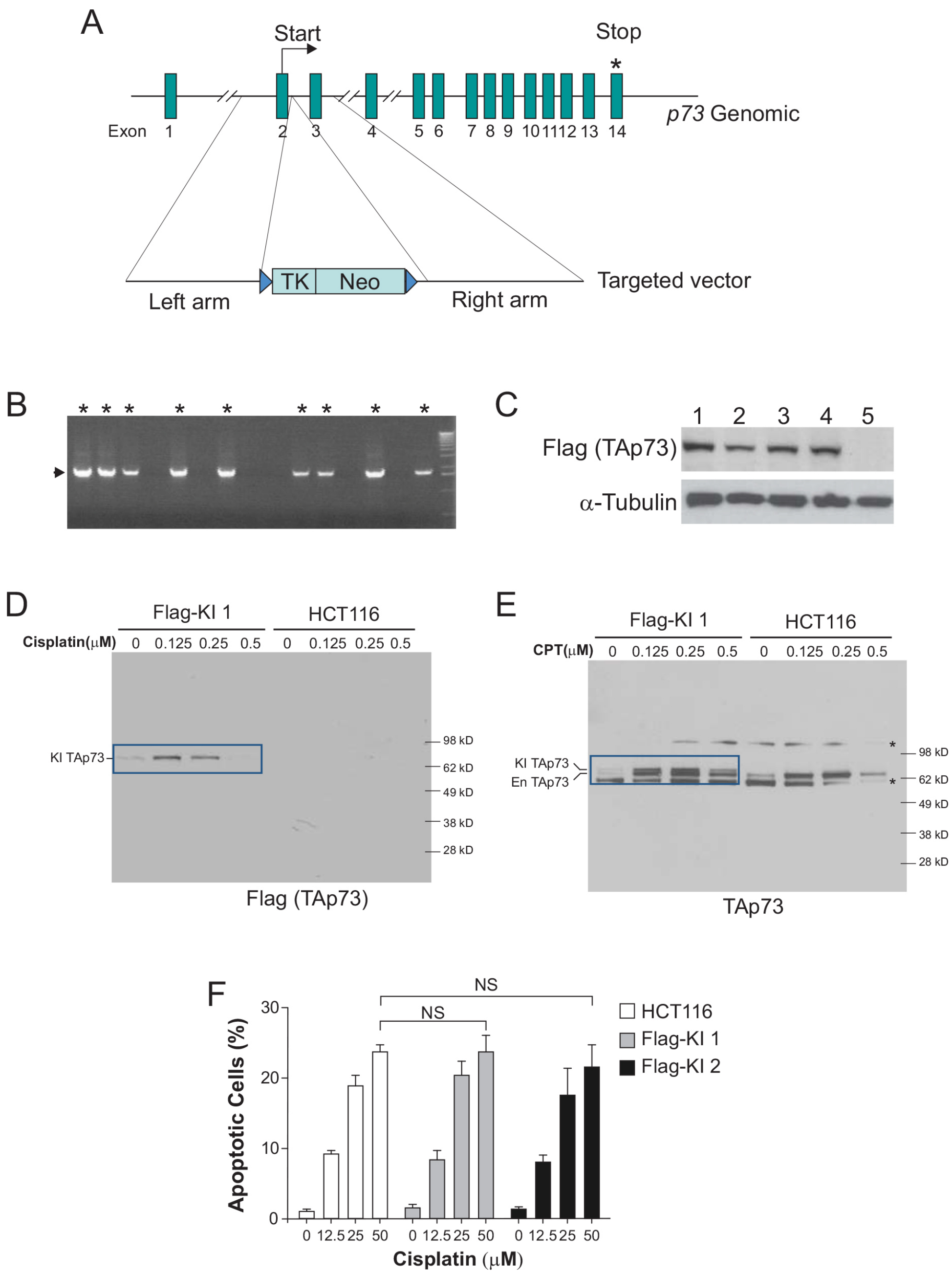

Supplementary Figure S1: Knock-in of Flag-tagged TAp73 in HCT116 cells. (A) Schematic representation of the genomic region of *p73* and the targeting vector for knocking in a triple-Flag tag in *TAp73*. **(B)** PCR screening of single G418-resistant HCT116 clones for gene targeting. * indicates positive clones. **(C)** Western blot analysis of Flag-tagged TAp73 expression in 4 example positive HCT116 clones and one negative sister clone. **(D)** Parental and Flag-KI HCT116 cells treated with cisplatin at indicated concentrations for 24 hr were probed for TAp73 by Flag western blotting. Square indicates the cropped portion shown in Fig. 1A. **(E)** Parental and Flag-KI HCT116 cells treated with camptothecin (CPT) at indicated concentrations for 24 hr were probed for TAp73 by p73 western blotting. Endogenous (En) and knock-in (KI) TAp73 are indicated. Square indicates the cropped portion shown in Fig. 1A, and * denotes non-specific bands. **(F)** Parental HCT116 cells and two independent clones with knock-in of Flag-tagged TAp73 (Flag-KI) were treated with cisplatin at indicated concentrations for 24 hr. Apoptosis was analyzed by counting cells with condensed and fragmented nuclei following staining with Hoechst 33258. Results were expressed as means ± s.d. of three independent experiments. NS, not significant.

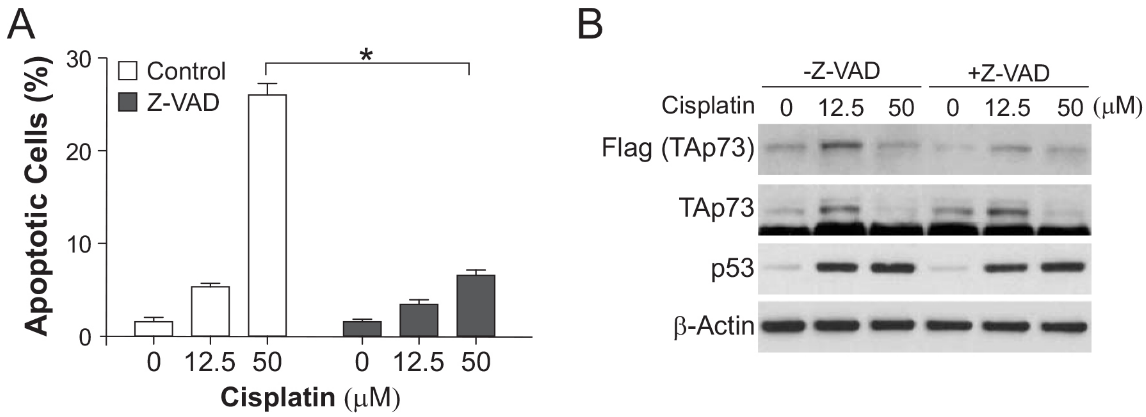

Supplementary Figure S2: Downregulation of TAp73 in response to extensive DNA damage is not a consequence of apoptosis. HCT116 cells were treated with cisplatin at indicated concentrations along with the pan-caspase inhibitor z-VAD-fmk (Z-VAD) for 24 hr. **(A)** Apoptosis was analyzed by nuclear staining. **(B)** Analysis of indicated proteins by western blotting.

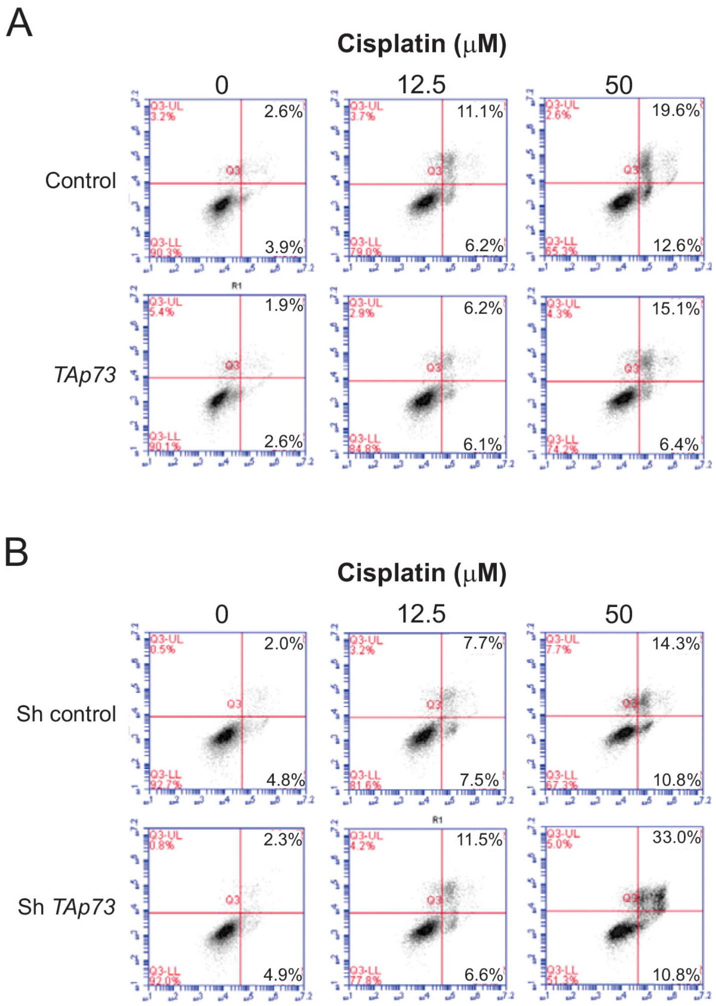

Supplementary Figure S3: Analysis of the effects of TAp73 transfection or knockdown on cisplatin-induced apoptosis by annexin V/propidium iodide (PI) staining. (A) HCT116 cells with Flag-TAp73 KI were transfected with control or HA-TAp73α construct, and then treated with cisplatin at indicated concentrations for 24 hr. After treatment, cells were stained with annexin V/PI and analyzed by flow cytometry. **(B)** HCT116 with stable transfection of *TAp73* or control shRNA were treated and analyzed as in (A). Two right quadrants in each plot represent annexin V-positive apoptotic cells, and their percentages are indicated.

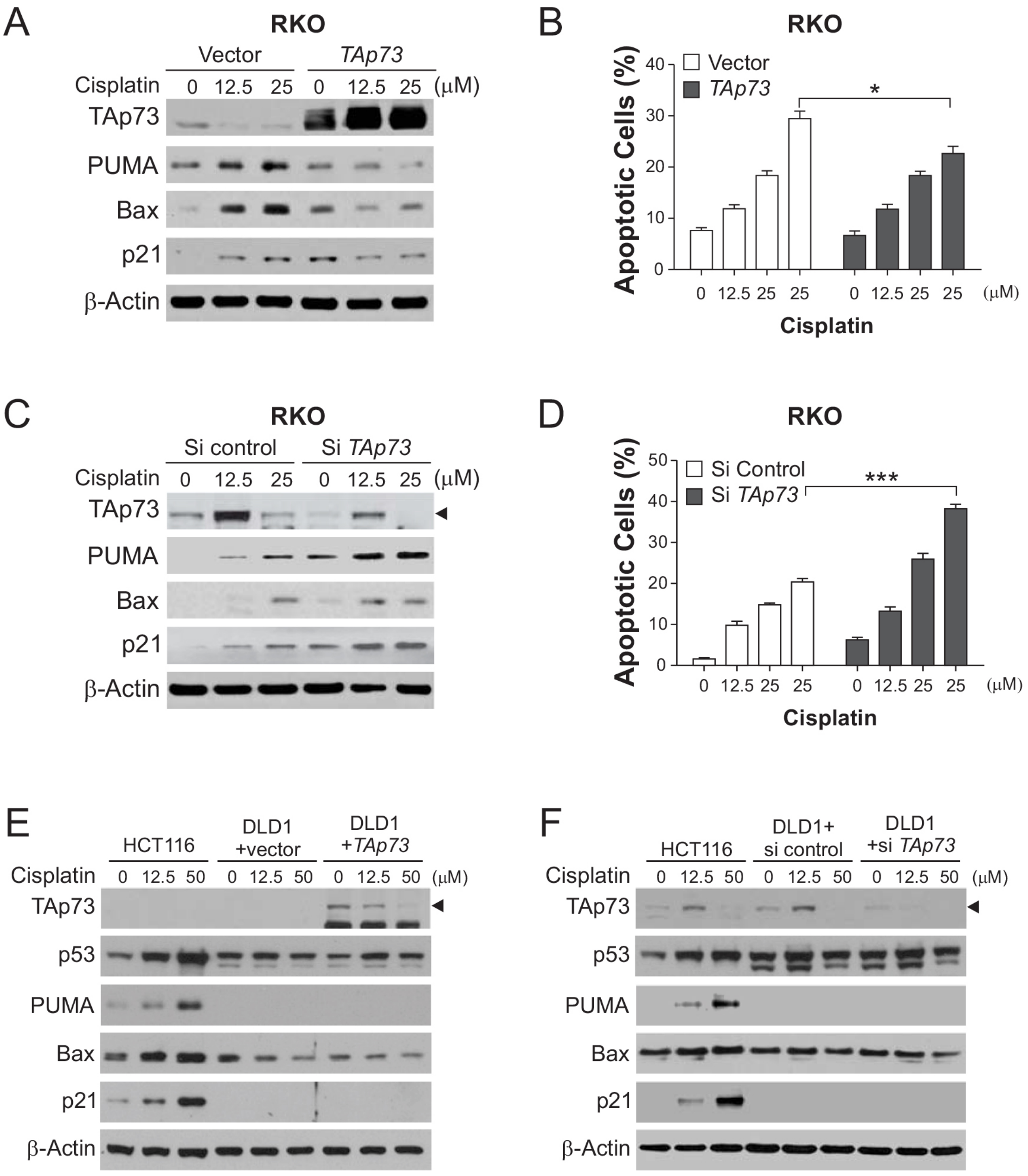

Supplementary Figure S4: TAp73 suppresses apoptosis and the expression of p53 downstream target genes induced by extensive DNA damage. (A) *p53*-WT RKO cells were transfected with control or HA-TAp73α construct, and then treated with cisplatin at indicated concentrations for 24 hr. Indicated proteins were analyzed by western blotting. **(B)** Apoptosis in cells treated as in (A) was analyzed by nuclear staining. **(C)** RKO cells were transfected with control or *TAp73* siRNA, and then treated with cisplatin at indicated concentrations for 24 hr. Indicated proteins were analyzed by western blotting. **(D)** Apoptosis in cells treated as in (C) was analyzed by nuclear staining. **(E)** *p53*-mutant DLD1 colon cancer cells were transfected with control or HA-TAp73α expression construct, and then treated with cisplatin at indicated concentrations for 24 hr. Indicated proteins were analyzed by western blotting. Lysates of HCT116 cells were loaded as control. **(F)** DLD1 cells were transfected with control or *TAp73* siRNA, and then treated with cisplatin at indicated concentrations for 24 hr. Indicated proteins were analyzed by western blotting. Lysates of HCT116 cells were loaded as control. Results in (B) and (D) were expressed as means ± s.d. of three independent experiments. ***, $P<0.001$; *, $P<0.05$.

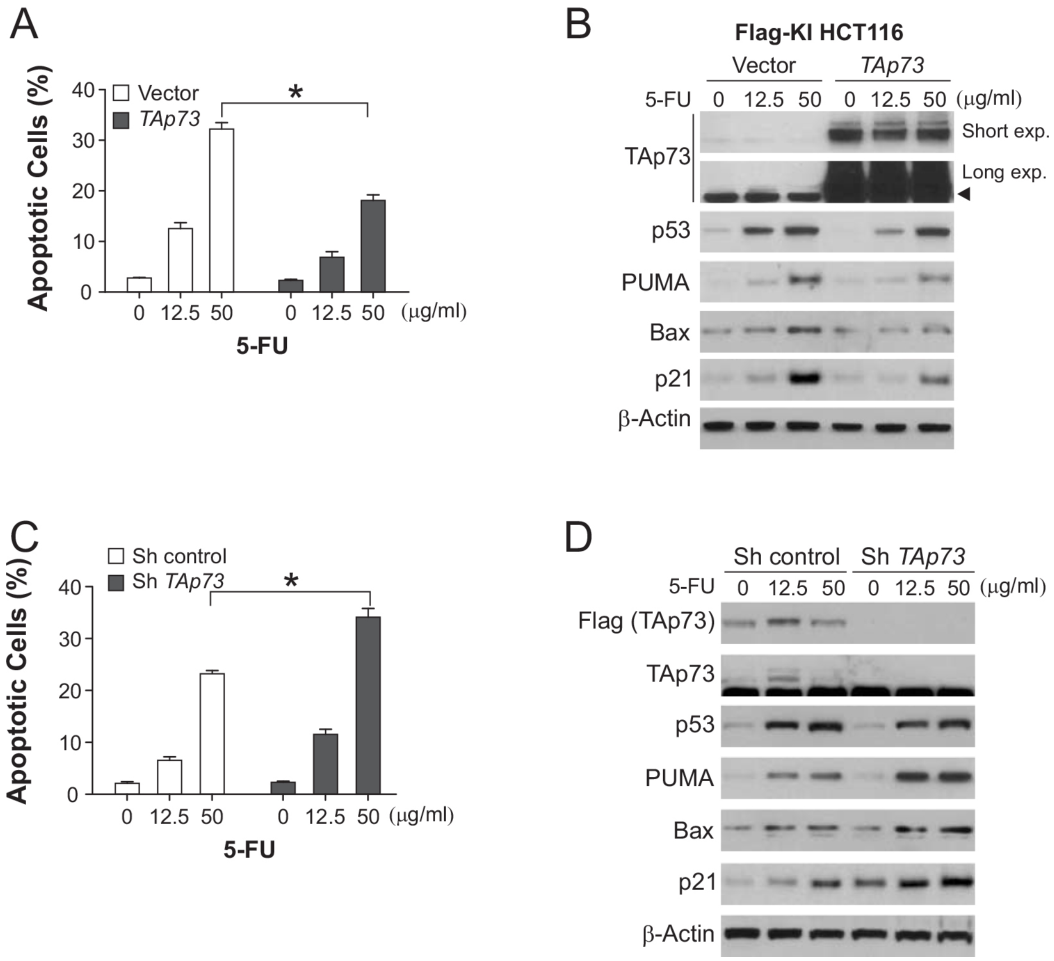

Supplementary Figure S5: Effects of TAp73 on 5-FU-induced apoptosis and p53 target gene expression. (A) HCT116 cells with Flag-TAp73 KI were transfected with control or HA-TAp73α construct, and then treated with 5-FU at indicated concentrations for 24 hr. Apoptosis was analyzed by nuclear staining. **(B)** Following treatment as in (A), indicated proteins were analyzed by western blotting. **(C)** HCT116 cells with stable transfection of *TAp73* or control shRNA were treated with 5-FU at indicated concentrations for 24 hr. Apoptosis was analyzed by nuclear staining. **(D)** Following treatment as in (C), indicated proteins were analyzed by western blotting. Results in (A) and (C) were expressed as means ± s.d. of three independent experiments. *****, $P < 0.05$.

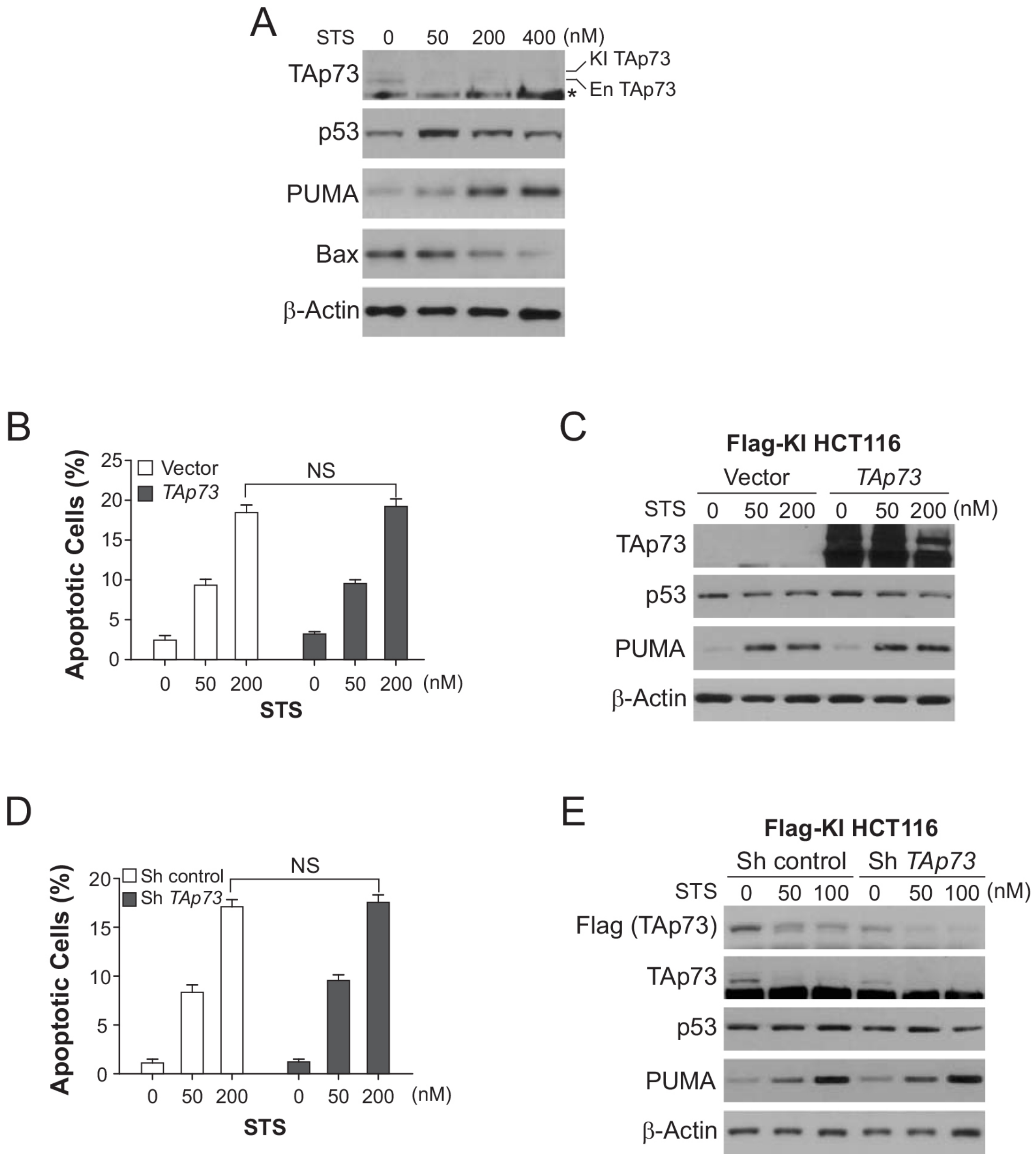

Supplementary Figure S6: Effects of TAp73 on staurosporine-induced apoptosis and p53 target gene expression. (A) HCT116 cells with Flag-TAp73 KI were treated with staurosporine (STS) at indicated concentrations for 24 hr. Indicated proteins were analyzed by western blotting. KI TAp73 and En TAp73 indicate knock-in and endogenous forms of TAp73α, respectively. * indicates non-specific bands detected by the p73 antibody. **(B)** HCT116 cells with Flag-TAp73 KI were transfected with control or HA-TAp73α construct, and then treated with STS at indicated concentrations for 24 hr. Apoptosis was analyzed by nuclear staining. **(C)** Following treatment as in (B), indicated proteins were analyzed by western blotting. **(D)** HCT116 cells with stable transfection of *TAp73* or control shRNA were treated with STS at indicated concentrations for 24 hr. Apoptosis was analyzed by nuclear staining. **(E)** Following treatment as in (D), indicated proteins were analyzed by western blotting. Results in (B) and (D) were expressed as means ± s.d. of three independent experiments. NS, not significant.

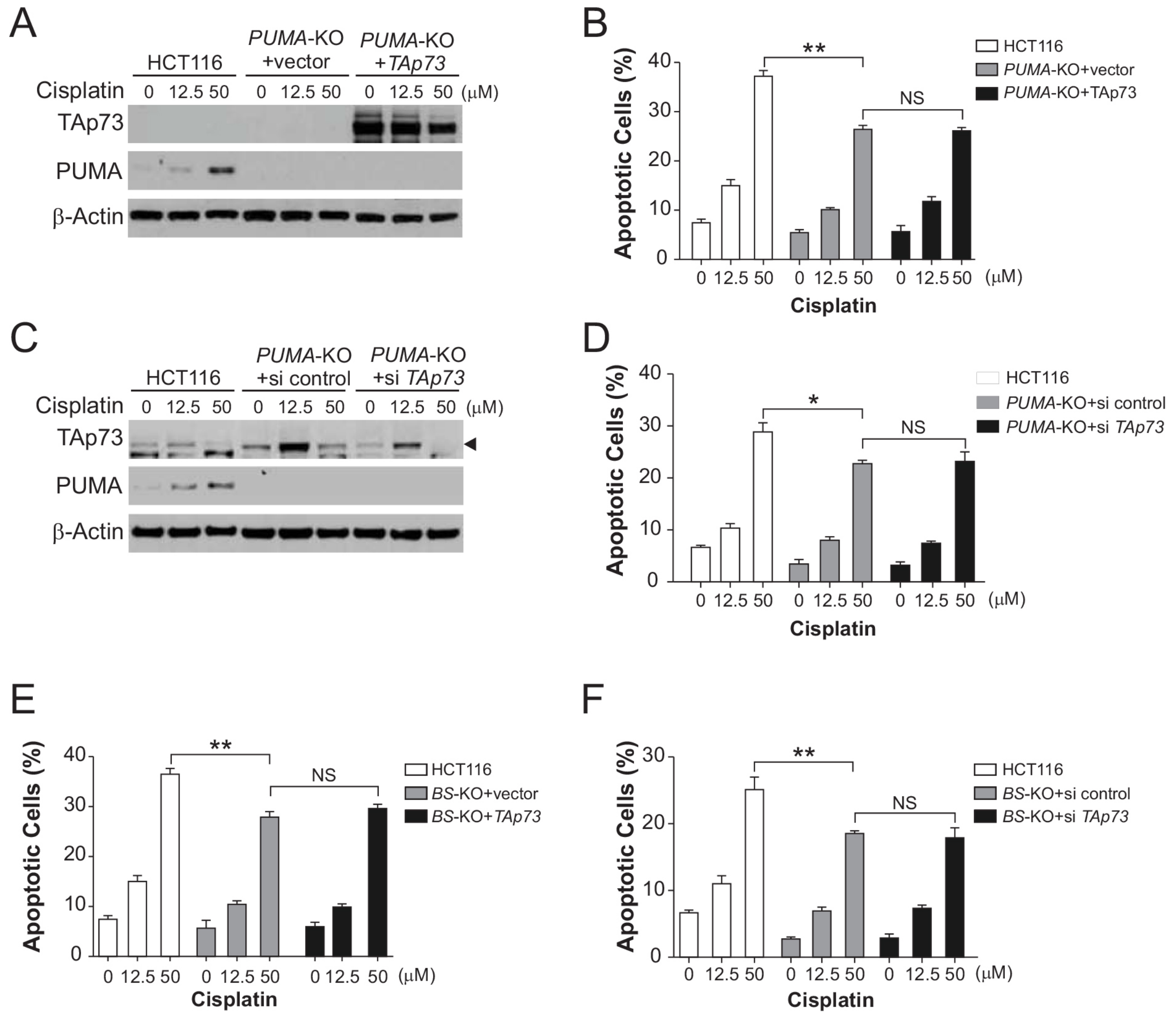

Supplementary Figure S7: TAp73 affects DNA damage-induced apoptosis through PUMA. **(A)** *PUMA*-knockout (*PUMA*-KO) HCT116 cells were transfected with control or TAp73α expression construct, and then treated with cisplatin at indicated concentrations for 24 hr. Indicated proteins were analyzed by western blotting. Lysates of HCT116 cells were loaded as control. **(B)** Apoptosis in cells treated as in (A) was analyzed by nuclear staining. **(C)** *PUMA*-KO HCT116 cells were transfected with control or *TAp73* siRNA, and then treated with cisplatin at indicated concentrations for 24 hr. Indicated proteins were analyzed by western blotting. Lysates of HCT116 cells were loaded as control. **(D)** Apoptosis in cells treated as in (C) was analyzed by nuclear staining. **(E)** HCT116 cells with the knockout of the p53 binding sites in the *PUMA* promoter (*BS*-KO) were transfected with control or TAp73α expression construct, and then treated with cisplatin at indicated concentrations for 24 hr. Apoptosis was analyzed by nuclear staining. **(F)** *BS*-KO cells were transfected with control or *TAp73* siRNA, and then treated with cisplatin at indicated concentrations for 24 hr. Apoptosis was analyzed by nuclear staining. Results in (B), (D), (E) and (F) were expressed as means ± s.d. of three independent experiments. **, *P* <0.01; *, *P* <0.05; NS, not significant.

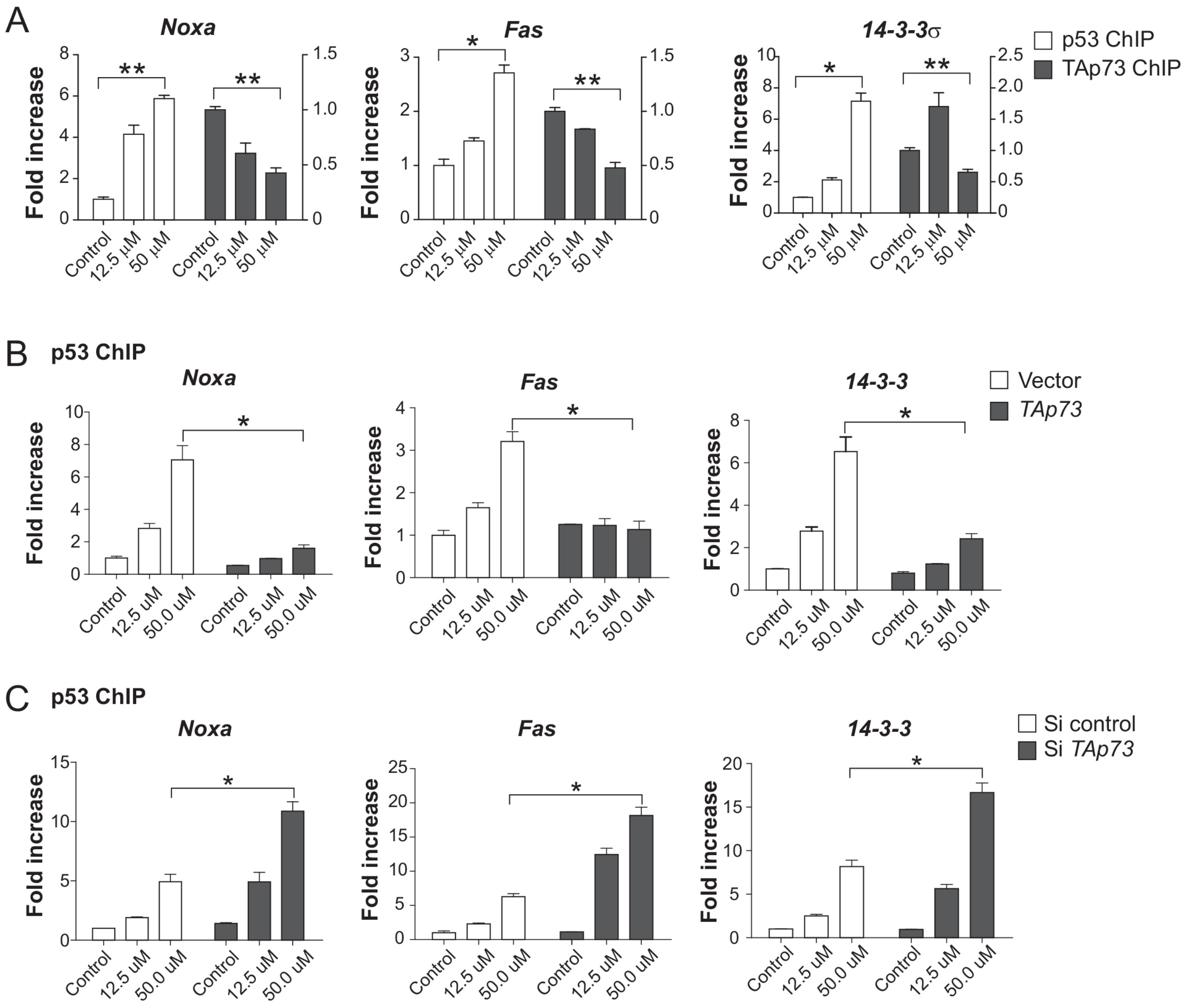

Supplementary Figure S8: Binding of p53 and TAp73 to different p53 responsive elements in response to different extents of DNA damage. (A) Flag-KI HCT116 cells were treated with cisplatin at indicated concentrations for 24 hr. The binding of p53 and TAp73 to the indicated promoters was analyzed by ChIP using anti-p53 antibody and anti-Flag-conjugated beads, respectively, followed by quantitative real-time PCR analysis. Results were normalized to those of IgG (for p53 ChIP) and parental HCT116 (for TAp73 ChIP), which were used as negative controls for ChIP, and plotted as fold of enrichment relative to control. **(B)** Flag-KI HCT116 cells were transfected with control or HA-TAp73α construct, and then treated with cisplatin at indicated concentrations for 24 hr. Occupancy of the indicated promoters by p53 was analyzed by ChIP as in (A). **(C)** Flag-KI HCT116 cells were transfected with control or *TAp73* siRNA, and then treated with cisplatin at indicated concentrations for 24 hr. Binding of p53 to the indicated promoters was analyzed as in (A). Results in (A)-(C) were expressed as means ± s.d. of three independent experiments. **, *P* <0.01; *, *P* <0.05.

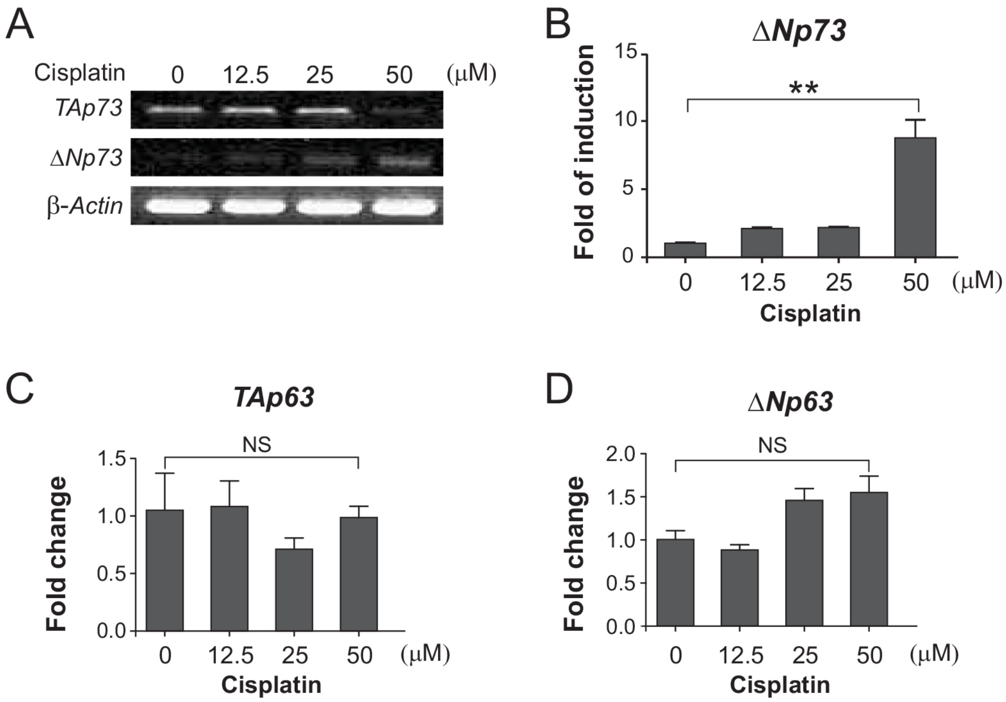

Supplementary Figure S9: mRNA expression of *p73* and *p63* isoforms in cells treated with cisplatin at different concentrations. (A) HCT116 cells were treated with cisplatin at indicated concentrations for 24 hr. *TAp73* and *ΔNp73* mRNA expression was analyzed by RT-PCR followed by agarose gel electrophoresis. **(B)-(D)** Following treatment as in (A), *ΔNp73* (B), *TAp63* (C), and *ΔNp63* (D) mRNA expression was analyzed by real-time RT-PCR and normalized to that of *β-actin*. **, *P* <0.01; NS, not significant.

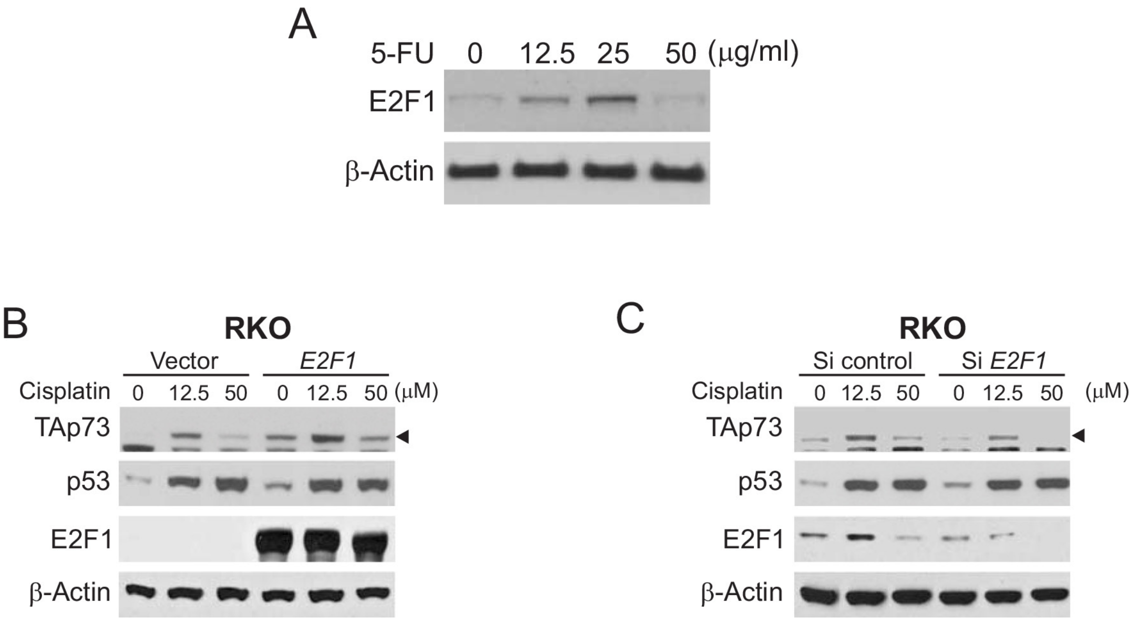

Supplementary Figure S10: Role of E2F1 in regulating TAp73 in response to lethal DNA damage. **(A)** HCT116 cells were treated with 5-FU at indicated concentrations for 24 hr. E2F1 expression was analyzed by western blotting. **(B)** RKO cells were transfected with control or E2F1 expression construct, and then treated with cisplatin at indicated concentrations for 24 hr. Indicated proteins were analyzed by western blotting. **(C)** RKO cells were transfected with control or *E2F1* siRNA, and then treated with cisplatin at indicated concentrations for 24 hr. Indicated proteins were analyzed by western blotting.

Supplementary Table S1. PCR primers

Experiment	Forward primer (5′ to 3′)	Reverse primer (5′ to 3′)
**Flag KI screening**	*CATGACGGTGATTATAAAGATC ATGACATC*	*GTGGATCTCGGCCTCCGTGAACTC CTCCTT*
**ChIP PCR**		
PUMA	*GTCGGTCTGTGTACGCATCG*	*AGACACCGGGACAGTCGGACAC*
p21	GTGGCTCTGATTGGCTTTCTG	CTGAAAACAGGCAGCCCAAG
Bax	AAAGCTCAGAGGCCCAAAAT	AGGCTGAGACGGGGTTATCT
14-3-3σ	CATTTAGGCAGTCTGATTCC	GCTCACGCCTGTCATCTC
Noxa	ACGATGTTCTTTCTGGCTGG	GCTTTGACCATCTGCAAACG
Fas	GAATTGAAGCGGAAGTCTGG	TGAGCAATATCTGTTCTGAAGG
TAp73	TGAGCCATGAAGATGTGCGAG	GCTGCTTATGGTCTGATGCTTATGG
GAPDH	GTATTCCCCCAGGTTTACAT	TTCTGTCTTCCACTCACTCC
**RT-PCR**		
E2F1	AGATGGTTATGGTGATCAAAGCC	ATCTGAAAGTTCTCCGAAGAGTCC
TAp73	GCACCACGTTTGAGCACCTCT	GCAGATTGAACTGGGCCATGA
ΔNp73	CAAACGGCCCGCATGTTCCC	TGGTCCATGGTGCTGCTCAGC
TAp63	AAGATGGTGCGACAAACAAG	AGAGAGCATCGAAGGTGGAG
ΔNp63	GGAAAACAATGCCCAGACTC	GTGGAATACGTCCAGGTGGC
p21	AGCGGAACAAGGAGTCAG	CGTTAGTGCCAGGAAAGAC
Bax	GGGTTGTCGCCCTTTTCTACTT	AGCCCATGATGGTTCTGATCAG
β-Actin	GACCTGACAGACTACCTCAT	AGACAGCACTGTGTTGGCTA
